# Regulation of Spiral Ganglion Neuron Regeneration as a Therapeutic Strategy in Sensorineural Hearing Loss

**DOI:** 10.3389/fnmol.2021.829564

**Published:** 2022-01-20

**Authors:** Man Wang, Lei Xu, Yuechen Han, Xue Wang, Fang Chen, Junze Lu, Haibo Wang, Wenwen Liu

**Affiliations:** Department of Otolaryngology-Head and Neck Surgery, Shandong Provincial ENT Hospital, Cheeloo College of Medicine, Shandong University, Jinan, China

**Keywords:** hearing loss, spiral ganglion neurons, regeneration, stem cells transplantation, glial cells

## Abstract

In the mammalian cochlea, spiral ganglion neurons (SGNs) are the primary neurons on the auditory conduction pathway that relay sound signals from the inner ear to the brainstem. However, because the SGNs lack the regeneration ability, degeneration and loss of SGNs cause irreversible sensorineural hearing loss (SNHL). Besides, the effectiveness of cochlear implant therapy, which is the major treatment of SNHL currently, relies on healthy and adequate numbers of intact SGNs. Therefore, it is of great clinical significance to explore how to regenerate the SGNs. In recent years, a number of researches have been performed to improve the SGNs regeneration strategy, and some of them have shown promising results, including the progress of SGN regeneration from exogenous stem cells transplantation and endogenous glial cells’ reprogramming. Yet, there are challenges faced in the effectiveness of SGNs regeneration, the maturation and function of newly generated neurons as well as auditory function recovery. In this review, we describe recent advances in researches in SGNs regeneration. In the coming years, regenerating SGNs in the cochleae should become one of the leading biological strategies to recover hearing loss.

## Introduction

The first world report of hearing released in 2021 warned that nearly 2.5 billion people, or one in four people in the world, will be living with some degree of hearing loss by 2050. Hearing loss affects many aspects of life, including communication, cognition, education, and even mental health, with significant detrimental effects economically on society. Among all hearing loss, sensorineural hearing loss (SNHL) is the most common type and accounts for the vast majority of them. The inner ear core parts are composed of hair cells (HCs) and spiral ganglion neurons (SGNs). The HCs function in transducing the sound mechanical stimulation into the primary acoustic signals (Liu Y. et al., 2019; Zhou et al., 2020), while the SGNs are primary afferent neurons in the auditory conduction pathway, and they transmit acoustic signals from the inner HCs of the cochlea to the central cochlear nucleus in the brainstem (Liu et al., 2021; Wang et al., 2021; Wei et al., 2021). Both HCs and SGNs can be injured by excessive noise exposure (Liberman, 2017; Guo L. et al., 2021), ototoxic drugs (Lang et al., 2005; He et al., 2017; Liu W. et al., 2019), aging (Bao and Ohlemiller, 2010; Kujawa and Liberman, 2015; He et al., 2021), genetic factors (Lv et al., 2021) and infections (Zhang et al., 2021), and thus leading to the SNHL.The SGNs in the cochlea can be divided into two subgroups-type I SGNs and type II SGNs, by their morphological structure, cell body, myelin sheath, and biological function. Type I SGNs, which account for the majority of auditory neurons in the inner ear, are myelinated, large, bipolar cells and primarily innervate the inner HCs, while the type II SGNs are unmyelinated, small, and innervate the outer HCs. More recently, single-cell profiling has been performed and three subpopulations of type I SGNs have been identified on the basis of transcriptional profiling (Shrestha et al., 2018; Sun et al., 2018). At the developmental level, the SGNs belong to terminally differentiated cells and can hardly regenerate by self-proliferation spontaneously and thus the damage of SGNs results in permanent SNHL (Guo et al., 2016, 2020). Besides, cochlear implant, which is the main clinical procedure for SNHL treatment, plays the role of restoring hearing by giving direct electrical stimulation to the viable SGNs (Guo et al., 2019, 2021b), and the effectiveness of cochlear implant therapy relies on healthy and adequate numbers of intact SGNs.

Therefore, prompting the strategies for regenerating SGNs is of great clinical significance for further advances in deafness treatment. In recent years, important advances have been made in a better understanding of the mechanisms involved in the regeneration of SGNs, which is a significant step toward the ultimate goal of improved hearing. In this review, we summarize the new discoveries about SGNs regeneration in recent years.

## Stem Cells Derived from Exogenous Sources Differentiate Toward SGNs

With the development of the inner ear after birth, the neuronal stem cell characteristics of the original stem cells continue to decline, and it lacks the endogenous cellular source for SGNs regeneration in the adult inner ear. Exogenous stem cells transplantation is an attractive alternative for adult SGNs regeneration. In recent years, studies have shown that many kinds of stem cells derived from exogenous sources, such as the induced pluripotent stem cells (iPSCs; Chen et al., 2017), mesenchymal stem cells (MSCs; Cho et al., 2011; Kil et al., 2016), and neural stem cells (NSCs; He et al., 2014), transplanted into the cochlea *in vivo* or *in vitro* could be differentiated toward SGNs. In addition, as the unique pluripotent cells, embryonic stem cells (ESCs) possess great differentiation potential and can differentiate into almost all cell types that make up the body. ESCs from the mouse or humans have been transplanted into the inner ear to replace the damaged SGNs (Corrales et al., 2006; Reyes et al., 2008; Hackelberg et al., 2017). Chang et al. (2021) found successful survival and migration of transplanted ESCs in the cochlea, and the transplanted ESC cells increased the auditory connection to the central auditory pathway in the hearing loss mice model.

Due to the cochlea’s special cavity-like structure, delivery of therapeutics to the inner ear becomes complicated due to their inaccessible location (Nyberg et al., 2019). The efficiency of regenerating SGNs and the survival of newly generated neurons are related to the transplantation method of exogenous stem cells, and thus it is necessary to find a suitable route for stem cell transplantation. Recently, transplantation through a small hole drilled into the scala tympani (ST), a fluid-filled lumen adjacent to the cochlear duct epithelium, has been widely used for its technically easier implementation (Hu et al., 2005; Lee et al., 2017; Chang et al., 2020). Besides, stem cell transplantation through the cochlear lateral wall was also found to be precise and safe, which has more efficacy to enter the Rosenthal’s canal (RC) compared with transplantation *via* the ST (Zhang et al., 2013).

Novel scaffolds could contribute to regulating neural stem cell proliferation, differentiation, and the oriented growth of derived neurons (Liu et al., 2018; Yan et al., 2018; Xia et al., 2020; Guo et al., 2021a). Hackelberg et al. (2017) present novel nanofibrous scaffolds for the guidance of stem cell-derived neurons for auditory nerve regeneration. The human ESC-derived neural precursor cells (NPC) implanted into the aligned nanofiber mats were efficiently differentiated into glutamatergic neurons and were guided into their target location of the cochlea in deafened guinea pigs. However, no improvement in eABR thresholds, or any functional improvement was found in mice implanted scaffolds with NPC compared with the cell-free (without NPC) scaffolds transplantation group (Hackelberg et al., 2017).

Collectively, researches have shown that the stem cells transplanted into cochlea could survive and acquiresome neuronal features, thus suggesting stem cell-based therapy might be a promising approach for auditory nerve regeneration. However, solid evidences for significant auditory function recovery after stem cell transplantation are still very limited (Fu et al., 2009; Chen et al., 2012, 2017; Hackelberg et al., 2017; Chang et al., 2021), and whether these exogenous cells can differentiate into functional SGNs and promote hearing recovery remain an open question. Meanwhile, proper integration of exogenous cells into the auditory circuit remains a fundamental challenge, and there are still some inevitable risks of transplanting stem cells into the inner ear, such as tumor formation, immune response (Nishimura et al., 2012), as well as ethical arguments regarding ESC cells transplantation. It is still necessary to develop safe and effective stem cell-based therapy for the clinical monitoring of cell transplantation.

## Glial Cells within The Inner Ear Are Potent Progenitors for SGN Reprogramming

**Figure 1:**
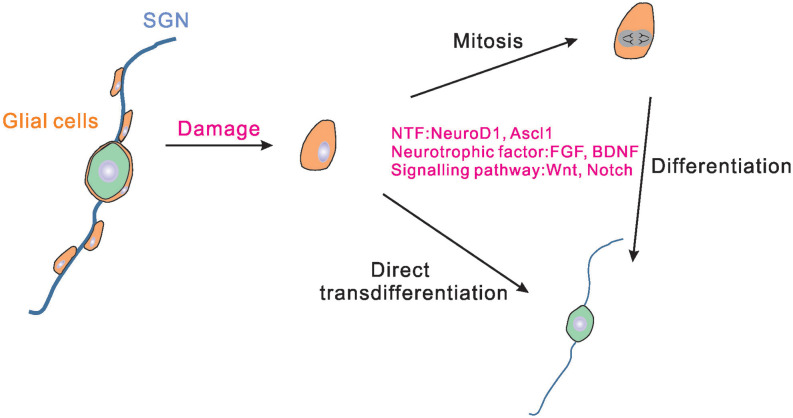
Schematic drawing of the endogenous glial cells regenerating SGNs. In the cochlea, satellite glial cells exist around SGNs. Satellite glial cells may proliferate and differentiate into SGNs when the SGNs are damaged. Some of the glial cells can also be directly transdifferentiated into SGNs. Neurogenic transcription factors, neurotrophic factors, and signal pathways could regulate the process of glial cells proliferation and differentiation. NTF, neurogenic transcription factors; SGNs, spiral ganglion neurons.

In the central nervous system (CNS), glial cells have been proved to be able to reprogram into functional neurons directly (Heins et al., 2002; Heinrich et al., 2010). Glial cells in the inner ear consist of Schwann cells that distribute along the fibers of SGNs and satellite glial cells that reside in RC surrounding SGNs cell bodies. It has been reported that glial cells in the inner ear play an irreplaceable role in protecting SGNs from degeneration and helping the SGNs to perform normal functions (Liu et al., 2014; Akil et al., 2015). The Schwann cells along the neuronal fibers express multiple neurotrophins, including the brain-derived neurotrophic factor (BDNF) and neurotrophin-3 (NT-3) that induce the development and survival of SGNs (Hansen et al., 2001). Recent studies found that the abundant glial cells in the inner ear could also become a promising resource for SGNs regeneration. McLean et al. (2016) reported that the separated proteolipid protein (PLP1) positive glial cells derived from the newborn mouse spiral ganglion could give rise to multiple cell types *in vitro*, including glial cells and neurons, and thus the PLP1 positive glial cells existing in the inner ear were identified as neural progenitors. Li X. et al. (2020) also found that glial cells in the mice inner ears started to express SGNs markers *in vivo* 6 days after Neurog1 (Ngn1) and Neurod1 ectopic expression. Moreover, a part of the newly generated SGNs exhibited a similar cellular phenotype (such as large round somas) to that of native SGNs and these SGNs could survive until postnatal day 42 (Li X. et al., 2020). In addition, it was found that even the spiral glial cells separated from adult human or rodent cochleae cultured *in vitro* could produce neurospheres and were capable of differentiating into neurons (Rask-Andersen et al., 2005; Lang et al., 2015). These studies revealed the stem cell potential of glial cells in the inner ear (Figure 1). Single cell RNA-sequencing analysis showed that satellite glial cells in the inner ear share many characteristics with CNS astrocytes (Tasdemir-Yilmaz et al., 2021), suggesting the important role of satellite glial cells in SGN regeneration. However, because of lack of an effective method for separating Schwann cells and satellite glial cells in the inner ear, the respective function of Schwann cells and satellite glial cells in SGNs regeneration remains to be defined.

Under normal conditions, the proliferation and regeneration ability of glial cells in the inner ear are really weak. Therefore, it is necessary to find ways to improve the ability of glial cells to regenerate SGNs. Neurogenic transcription factors have been utilized widely in many studies trying to reprogram glial cells into SGNs. The current advanced researches on viral vectors make it more convenient and effective to use virus-mediated neurogenic transcription factors expression to reprogram glial cells into neurons. For example, Chen et al. (2020) demonstrated that adeno-associated virus-mediated NeuroD1 ectopic overexpression contributed to astrocyte-to-neuron conversion in adult mammalian brains. Through NeuroD1-based gene therapy, a good deal of functional new neurons were generated in the ischemic injured cortex and brain function was repaired after ischemic injury (Chen et al., 2020). In addition to NeuroD1 and Ngn1 (Li X. et al., 2020), Ascl1 was also proved to be able to reprogram the spiral ganglion cells into induced SGNs (Nishimura et al., 2014; Noda et al., 2018). It is worth noting that the combination of two factors results in better reprogramming of reactive glial cells to neurons than using one single copy alone (Figure 1).

## SGNs Damage Induces Glial Cells Proliferation

Activation of astrocytes (reactive gliosis) has been considered as the sign of a lesion to the nervous system in conditions such as mechanical damage to the brain or neurodegenerative diseases (de Melo et al., 2012; Pekny et al., 2014). Similar to the survival characteristics of glial cells in the CNS, glial cells in the inner ear also presented greater survival traits than SGNs (Wise et al., 2017). Wise et al. (2017) found that 6 weeks after deafness duration, the morphology of glial cells was altered following the SGNs soma loss. The soma of Schwann cell assumed an “astrocyte-like” morphology and dedifferentiation might have occurred in the Schwann cells. Studies showed that moderate ouabain administration could selectively cause SGNs damage and abolish hearing without influencing HCs (Lang et al., 2005; Yuan et al., 2013). This drug that specifically damages endogenous SGNs allows scientists to better focus on the responses of inner ear cells after SGNs damage. Lang et al. (2011) found that ouabain application to the round window caused a significant up-regulation in glial cells proliferation and increase of Sox2 protein expression, which is the marker of neural progenitor. In addition, after ouabain exposure, the morphology of glial cells nuclei also changed, becoming enlarged and rounded in shape, similar to the nuclei of SGNs (Lang et al., 2015). Furthermore, Kempfle et al. (2020) found that ouabain treatment in adult mouse inner ear induced SGNs damage and the transient overexpression of Lin28 in Plp1-positive glial cells led to increased proliferation and neural conversion. Thus, these results demonstrated that the damage of SGNs induced inner ear glial cells proliferation and morphological changes, while the proliferative glial cells may be poised to regenerate the auditory nerve when given appropriate stimuli.

## Neurotrophic Factors and Signaling Pathways Enhance The SGNs Regeneration

Some growth factors and neurotrophins, such as fibroblast growth factor (FGF), BDNF, glial cell-derived neurotrophic factor (GDNF), and NT-3, play important roles during SGNs development and survival (Johnson Chacko et al., 2017). Treatment of mature BDNF and proBDNF in SGNs cultures with a concentration as low as ng/ml-range could exert a protective effect on SGNs against degeneration (Schulze et al., 2020). Previous studies also found that FGF, GDNF, and NT-3 promoted the outgrowth of neurites from cultured SGNs, which indicated that these molecules may also have trophic functions in SGNs regeneration (Wei et al., 2007; Wang and Green, 2011; Garcia-Hernandez et al., 2013). Currently, a combination administration of stem cells and different growth factors or neurotrophic molecules was evolved in hearing loss studies. In *in vivo* experiments, transducing the mesenchymal cells within perilymphatic compartments of the cochlea with a recombinant plasmid gene that drives BDNF expression stimulated the regeneration of spiral ganglion neurites (Pinyon et al., 2014). With BDNF overexpression, the regenerated spiral ganglion neurites could extend to the area of cochlear implant electrodes, with localized ectopic branching (Pinyon et al., 2014). The success in regenerating SGNs in rodents by combined administration of neurotrophic factors with stem cells has led to great developments in cochlea implant technology (Scheper et al., 2019).

On the delivery system of neurotrophic factors, coating the artificial cochlea with ultra-high viscous alginate containing BDNF-overexpressing MSCs resulted in increased SGNs density in the inner ear of deafened animal (Scheper et al., 2019). In order to improve the survival of stem cells in the inner ear and increase the efficiency of SGNs generation, Chang et al. (2020) cultured human ESC-derived spheroids in an artificial three-dimensional (3D) microenvironment composed of specific hydrogel combined with a sustained release BDNF delivery system (PODS-hBDNF). The transplanted human ESC-derived otic neuronal progenitors spheroids survived and neuronally differentiated into otic neuronal lineages *in vitro* and *in vivo* and also extended neurites toward the bony wall of the cochlea (Chang et al., 2020). In some studies exploring the differentiation of glial cells into SGNs, neurotrophic factors are generally added to the culture medium to promote the regeneration of SGNs and maintain the survival of newly generated neurons (Diensthuber et al., 2014; McLean et al., 2016). In a word, supplementation of neurotrophic factors could enhance the regeneration of SGNs.

Moreover, modulation of some specific signaling pathways also promoted robust neuronal differentiation. For example, Zong et al. (2014) found that the Wnt signaling pathway plays a critical role in stimulating the differentiation of amniotic fluid-derived stem cells into functional neurons. The role of Wnt signaling in triggering neurogenesis in the gentamicin-lesioned cochlear cultures was also proved by Bas et al. (2014). Pharmacologic activation of Wnt/β-catenin pathway together with human nasal MSC treatment could induce robust differentiation of SGNs. In addition, Notch signaling also had a significant role in the inner ear stem cells fate decision. Different from the role in HCs differentiation, continued Notch signaling increased the expansion of neuronal progenitors and promoted progenitor cells to enter a neuronal lineage by directly increasing Ngn1 expression (Jeon et al., 2011). The PI3K/Akt signaling pathway was verified to be involved in promoting neuron differentiation in the inner ears (Zhang et al., 2016). Recently, Perny et al. (2017) developed a protocol based on 3D organoid culture systems, which guided mouse ESCs differentiating into otic sensory neurons by activating the bone morphogenetic protein (BMP) signaling and concomitantly inhibiting the transforming growth factor beta (TGFβ) signaling. In this study, BDNF and NT-3 were also supplemented into the culture to guide neuronal maturation (Perny et al., 2017). The neurotrophic factors and certain signaling pathways found to promote proliferation and neural differentiation may reveal the mechanisms underlying stem cell differentiation (Figure 1).

## Transcriptome Analysis and SGNs Regeneration

By comparing similarities and differences of transcriptional regulatory networks in multiple events during sensory cell development or regeneration, key factors or cell signaling pathways could be found to be involved in the modulation of cell regeneration (Atkinson et al., 2015). In 2016, Kwan introduced the single-cell transcriptome analysis application in SGNs regeneration (Kwan, 2016). Transcriptome profiling of cells at different stages of SGNs differentiation, including exogenous stem cell-derived and endogenous glial cell-derived SGNs regeneration, could provide useful information about the molecular processes involved. Most recently, the emerging cell transcriptome analysis such as single-cell RNA sequencing and bulk RNA-sequencing have been applied in identifying the subtypes of SGNs and inner ear glial cells, characterizing the dynamic expression pattern of SGN genes, and analyzing the transcriptome of the induced neurons after regeneration (Shrestha et al., 2018; Sun et al., 2018; Li C. et al., 2020; Chen et al., 2021; Tasdemir-Yilmaz et al., 2021). For example, Li C. et al. (2020) compared the transcriptomes of SGNs and two other inner ear cell types, HCs and glia to identify genes that were expressed specifically in SGNs within the cochlea and exhibited either constant (e.g., Scrt2) or dynamic (e.g., Celf4) expression patterns. These genes might represent promising candidate regulators of SGN cell-fate determination and/or differentiation. Chen et al. (2021) performed transcriptomic analysis of the glial cells-derived SGNs under different stimuli conditions *in vitro* and found that the small molecules cocktail FIBCL treatment promoted the newborn SGNs maturation. Despite the transcriptome analysis of the inner ear cells still facing some challenges such as a limited number of cells obtained from the cochleae, the lower cell viability, etc. it is predicted to play a great role in enhancing the SGNs regeneration research in the future.

## The Function of Newly Regenerated Neurons

In most of the *in vitro* studies about SGNs regeneration, the newly generated neurons were confirmed by neuronal morphology or by immunostaining of several neuronal markers, including TUJ1, MAP2, Prox1, Gata3, etc. (Diensthuber et al., 2014; Rousset et al., 2020). The electrophysiological characteristic function of newly generated neurons was also identified to determine whether newborn neurons have electrophysiological functions. For example, recording the whole-cell current of the newly generated neurons; using specific Na^+^ channel antagonist tetrodotoxin (TTX) to prove whether there are Na^+^ channels on the newborn neurons and recording the glutamate induced-current on neurons to confirm whether newborn neurons responding to the main neurotransmitter in the cochlea (Crozier and Davis, 2014; Davis and Crozier, 2015). In the induced newborn neurons, Nishimura et al. (2014) measured the dynamics of neuronal-like membrane potential. Ascl1 and NeuroD1 double overexpressed cochlear epithelial cells exhibit neuronal firing on days *in vitro* 8 and the inward membrane currents could be reduced by the treatment of TTX, which indicates the existence of a robust membrane Na^+^ current (Nishimura et al., 2014).

In *in vivo* study, functional analyses of the peripheral auditory system can be investigated using audiological tests. ABR is a reflection of the neural activity transmitted from the scalp that reflects synchronous neural activity within the auditory nerve, and subsequently to the brainstem. A previous study has shown that the ABR wave I latency and amplitude were related to the corresponding SGNs density and function (Li et al., 2016; Zhang et al., 2018).

## Conclusion and Future Perspectives

According to these foundational studies of SGNs regeneration in recent years, the reprogramming of exogenous stem cells or endogenous inner ear cells into SGNs seems to be a promising strategy. The application of some neurogenic transcription factors helps the glial cells proliferation and reprogramming. Combined transplantation of stem cells and neurotrophic factors enhances the effectiveness of SGNs regeneration and improves the survival of newly generated neurons. In addition, modulation of some signaling pathways also promotes neural differentiation. Although it is still necessary to clarify details about SGNs regeneration in humans, the early success of these interventions increases our expectation about the auditory nerve regeneration in human patients in the future.

Still, more efforts are needed to improve the effectiveness of SGNs regeneration, especially to promote the maturation of newly generated neurons. The newly generated SGNs are needed to not only present neuronal morphology and express neuronal protein markers, but also to possess electrophysiological functions, generate action potentials as well as form synaptic connections with HCs in the inner ear and with conductive neurons in the brain stem. As type I and type II SGNs perform different functions in the cochlea and the appearance of the three subtypes of type I SGN indicates the maturity of neurons, differentiating these cells into the desired mature subtypes is also crucial.

## Author Contributions

MW, LX, YH, XW, FC, JL, HW, and WL drafted and wrote the manuscript. All authors contributed to the article and approved the submitted version.

## Conflict of Interest

The authors declare that the research was conducted in the absence of any commercial or financial relationships that could be construed as a potential conflict of interest.

## Publisher’s Note

All claims expressed in this article are solely those of the authors and do not necessarily represent those of their affiliated organizations, or those of the publisher, the editors and the reviewers. Any product that may be evaluated in this article, or claim that may be made by its manufacturer, is not guaranteed or endorsed by the publisher.

## References

[B1] Akil O. Sun Y. Vijayakumar S. Zhang W. Ku T. Lee C. K.. (2015). Spiral ganglion degeneration and hearing loss as a consequence of satellite cell death in saposin B-deficient mice. J. Neurosci. 35, 3263–3275. 10.1523/JNEUROSCI.3920-13.2015 25698761 PMC6605599

[B2] Atkinson P. J. Huarcaya Najarro E. Sayyid Z. N. Cheng A. G. (2015). Sensory hair cell development and regeneration: similarities and differences. Development 142, 1561–1571. 10.1242/dev.114926 25922522 PMC4419275

[B3] Bao J. Ohlemiller K. K. (2010). Age-related loss of spiral ganglion neurons. Hear. Res. 264, 93–97. 10.1016/j.heares.2009.10.009 19854255 PMC2868093

[B4] Bas E. Van De Water T. R. Lumbreras V. Rajguru S. Goss G. Hare J. M.. (2014). Adult human nasal mesenchymal-like stem cells restore cochlear spiral ganglion neurons after experimental lesion. Stem Cells Dev. 23, 502–514. 10.1089/scd.2013.0274 24172073 PMC3928683

[B5] Chang H. T. Heuer R. A. Oleksijew A. M. Coots K. S. Roque C. B. Nella K. T.. (2020). An engineered three-dimensional stem cell niche in the inner ear by applying a nanofibrillar cellulose hydrogel with a sustained-release neurotrophic factor delivery system. Acta Biomater. 108, 111–127. 10.1016/j.actbio.2020.03.007 32156626 PMC7198367

[B6] Chang S. Y. Jeong H. W. Kim E. Jung J. Y. Lee M. Y. (2021). Distribution and afferent effects of transplanted mESCs on cochlea in acute and chronic neural hearing loss models. Biomed Res. Int. 2021:4956404. 10.1155/2021/4956404 34250085 PMC8238572

[B7] Chen J. Guan L. Zhu H. Xiong S. Zeng L. Jiang H. (2017). Transplantation of mouse-induced pluripotent stem cells into the cochlea for the treatment of sensorineural hearing loss. Acta Otolaryngol. 137, 1136–1142. 10.1080/00016489.2017.1342045 28643534

[B10] Chen Z. Huang Y. Yu C. Liu Q. Qiu C. Wan G. (2021). Cochlear Sox2(+) glial cells are potent progenitors for spiral ganglion neuron reprogramming induced by small molecules. Front. Cell Dev. Biol. 9:728352. 10.3389/fcell.2021.728352 34621745 PMC8490772

[B8] Chen W. Jongkamonwiwat N. Abbas L. Eshtan S. J. Johnson S. L. Kuhn S.. (2012). Restoration of auditory evoked responses by human ES-cell-derived otic progenitors. Nature 490, 278–282. 10.1038/nature11415 22972191 PMC3480718

[B9] Chen Y. C. Ma N. X. Pei Z. F. Wu Z. Do-Monte F. H. Keefe S.. (2020). A neuroD1 AAV-based gene therapy for functional brain repair after ischemic injury through *in vivo* astrocyte-to-neuron conversion. Mol. Ther. 28, 217–234. 10.1016/j.ymthe.2019.09.003 31551137 PMC6952185

[B11] Cho Y. B. Cho H. H. Jang S. Jeong H. S. Park J. S. (2011). Transplantation of neural differentiated human mesenchymal stem cells into the cochlea of an auditory-neuropathy guinea pig model. J. Korean Med. Sci. 26, 492–498. 10.3346/jkms.2011.26.4.492 21468255 PMC3069567

[B12] Corrales C. E. Pan L. Li H. Liberman M. C. Heller S. Edge A. S. (2006). Engraftment and differentiation of embryonic stem cell-derived neural progenitor cells in the cochlear nerve trunk: growth of processes into the organ of Corti. J. Neurobiol. 66, 1489–1500. 10.1002/neu.20310 17013931 PMC2040047

[B13] Crozier R. A. Davis R. L. (2014). Unmasking of spiral ganglion neuron firing dynamics by membrane potential and neurotrophin-3. J. Neurosci. 34, 9688–9702. 10.1523/JNEUROSCI.4552-13.2014 25031408 PMC4099546

[B14] Davis R. L. Crozier R. A. (2015). Dynamic firing properties of type I spiral ganglion neurons. Cell Tissue Res. 361, 115–127. 10.1007/s00441-014-2071-x 25567109

[B15] de Melo J. Miki K. Rattner A. Smallwood P. Zibetti C. Hirokawa K.. (2012). Injury-independent induction of reactive gliosis in retina by loss of function of the LIM homeodomain transcription factor Lhx2. Proc. Natl. Acad. Sci. U S A 109, 4657–4662. 10.1073/pnas.1107488109 22393024 PMC3311371

[B16] Diensthuber M. Zecha V. Wagenblast J. Arnhold S. Edge A. S. Stover T. (2014). Spiral ganglion stem cells can be propagated and differentiated into neurons and glia. Biores. Open Access 3, 88–97. 10.1089/biores.2014.0016 24940560 PMC4048968

[B17] Fu Y. Wang S. Liu Y. Wang J. Wang G. Chen Q.. (2009). Study on neural stem cell transplantation into natural rat cochlea *via* round window. Am. J. Otolaryngol. 30, 8–16. 10.1016/j.amjoto.2007.12.006 19027507

[B18] Garcia-Hernandez S. Potashner S. J. Morest D. K. (2013). Role of fibroblast growth factor 8 in neurite outgrowth from spiral ganglion neurons *in vitro*. Brain Res. 1529, 39–45. 10.1016/j.brainres.2013.07.030 23891716 PMC5217747

[B19] Guo L. Cao W. Niu Y. He S. Chai R. Yang J. (2021). Autophagy regulates the survival of hair cells and spiral ganglion neurons in cases of noise, ototoxic drug and age-induced sensorineural hearing loss. Front. Cell. Neurosci. 15:760422. 10.3389/fncel.2021.760422 34720884 PMC8548757

[B20] Guo R. Li J. Chen C. Xiao M. Liao M. Hu Y.. (2021a). Biomimetic 3D bacterial cellulose-graphene foam hybrid scaffold regulates neural stem cell proliferation and differentiation. Colloids Surf. B Biointerfaces 200:111590. 10.1016/j.colsurfb.2021.111590 33529926

[B21] Guo R. Liao M. Ma X. Hu Y. Qian X. Xiao M.. (2021b). Cochlear implant-based electric-acoustic stimulation modulates neural stem cell-derived neural regeneration. J. Mater. Chem. B. 9, 7793–7804. 10.1039/d1tb01029h 34586130

[B22] Guo R. Ma X. Liao M. Liu Y. Hu Y. Qian X.. (2019). Development and application of cochlear implant-based electric-acoustic stimulation of spiral ganglion neurons. ACS Biomater. Sci. Eng. 5, 6735–6741. 10.1021/acsbiomaterials.9b01265 33423491

[B23] Guo R. Xiao M. Zhao W. Zhou S. Hu Y. Liao M.. (2020). 2D Ti_3_C_2_T_x_MXene couples electrical stimulation to promote proliferation and neural differentiation of neural stem cells. Acta Biomater. 10.1016/j.actbio.2020.12.035. [Online ahead of print]. 33348061

[B24] Guo R. Zhang S. Xiao M. Qian F. He Z. Li D.. (2016). Accelerating bioelectric functional development of neural stem cells by graphene coupling: implications for neural interfacing with conductive materials. Biomaterials 106, 193–204. 10.1016/j.biomaterials.2016.08.019 27566868

[B25] Hackelberg S. Tuck S. J. He L. Rastogi A. White C. Liu L.. (2017). Nanofibrous scaffolds for the guidance of stem cell-derived neurons for auditory nerve regeneration. PLoS One 12:e0180427. 10.1371/journal.pone.0180427 28672008 PMC5495534

[B26] Hansen M. R. Vijapurkar U. Koland J. G. Green S. H. (2001). Reciprocal signaling between spiral ganglion neurons and Schwann cells involves neuregulin and neurotrophins. Hear. Res. 161, 87–98. 10.1016/s0378-5955(01)00360-4 11744285

[B28] He Z. Guo L. Shu Y. Fang Q. Zhou H. Liu Y.. (2017). Autophagy protects auditory hair cells against neomycin-induced damage. Autophagy 13, 1884–1904. 10.1080/15548627.2017.1359449 28968134 PMC5788479

[B29] He Z. H. Li M. Fang Q. J. Liao F. L. Zou S. Y. Wu X.. (2021). FOXG1 promotes aging inner ear hair cell survival through activation of the autophagy pathway. Autophagy 17, 4341–4362. 10.1080/15548627.2021.1916194 34006186 PMC8726647

[B27] He Y. Zhang P. Z. Sun D. Mi W. J. Zhang X. Y. Cui Y.. (2014). Wnt1 from cochlear schwann cells enhances neuronal differentiation of transplanted neural stem cells in a rat spiral ganglion neuron degeneration model. Cell Transplant. 23, 747–760. 10.3727/096368913X669761 23809337

[B30] Heinrich C. Blum R. Gascon S. Masserdotti G. Tripathi P. Sanchez R.. (2010). Directing astroglia from the cerebral cortex into subtype specific functional neurons. PLoS Biol. 8:e1000373. 10.1371/journal.pbio.1000373 20502524 PMC2872647

[B31] Heins N. Malatesta P. Cecconi F. Nakafuku M. Tucker K. L. Hack M. A.. (2002). Glial cells generate neurons: the role of the transcription factor Pax6. Nat. Neurosci. 5, 308–315. 10.1038/nn828 11896398

[B32] Hu Z. Wei D. Johansson C. B. Holmstrom N. Duan M. Frisen J.. (2005). Survival and neural differentiation of adult neural stem cells transplanted into the mature inner ear. Exp. Cell Res. 302, 40–47. 10.1016/j.yexcr.2004.08.023 15541724

[B33] Jeon S. J. Fujioka M. Kim S. C. Edge A. S. (2011). Notch signaling alters sensory or neuronal cell fate specification of inner ear stem cells. J. Neurosci. 31, 8351–8358. 10.1523/JNEUROSCI.6366-10.2011 21653840 PMC3136123

[B34] Johnson Chacko L. Blumer M. J. F. Pechriggl E. Rask-Andersen H. Dietl W. Haim A.. (2017). Role of BDNF and neurotrophic receptors in human inner ear development. Cell Tissue Res. 370, 347–363. 10.1007/s00441-017-2686-9 28924861

[B35] Kempfle J. S. Luu N. C. Petrillo M. Al-Asad R. Zhang A. Edge A. S. B. (2020). Lin28 reprograms inner ear glia to a neuronal fate. Stem Cells 38, 890–903. 10.1002/stem.3181 32246510 PMC10908373

[B36] Kil K. Choi M. Y. Park K. H. (2016). *In vitro* differentiation of human wharton’s jelly-derived mesenchymal stem cells into auditory hair cells and neurons. J. Int. Adv. Otol. 12, 37–42. 10.5152/iao.2016.1190 27340981

[B37] Kujawa S. G. Liberman M. C. (2015). Synaptopathy in the noise-exposed and aging cochlea: primary neural degeneration in acquired sensorineural hearing loss. Hear. Res. 330, 191–199. 10.1016/j.heares.2015.02.009 25769437 PMC4567542

[B38] Kwan K. Y. (2016). Single-cell transcriptome analysis of developing and regenerating spiral ganglion neurons. Curr. Pharmacol. Rep. 2, 211–220. 10.1007/s40495-016-0064-z 28758056 PMC5531199

[B39] Lang H. Li M. Kilpatrick L. A. Zhu J. Samuvel D. J. Krug E. L.. (2011). Sox2 up-regulation and glial cell proliferation following degeneration of spiral ganglion neurons in the adult mouse inner ear. J. Assoc. Res. Otolaryngol. 12, 151–171. 10.1007/s10162-010-0244-1 21061038 PMC3046328

[B40] Lang H. Schulte B. A. Schmiedt R. A. (2005). Ouabain induces apoptotic cell death in type I spiral ganglion neurons, but not type II neurons. J. Assoc. Res. Otolaryngol. 6, 63–74. 10.1007/s10162-004-5021-6 15735933 PMC2504640

[B41] Lang H. Xing Y. Brown L. N. Samuvel D. J. Panganiban C. H. Havens L. T.. (2015). Neural stem/progenitor cell properties of glial cells in the adult mouse auditory nerve. Sci. Rep. 5:13383. 10.1038/srep13383 26307538 PMC4549618

[B42] Lee M. Y. Hackelberg S. Green K. L. Lunghamer K. G. Kurioka T. Loomis B. R.. (2017). Survival of human embryonic stem cells implanted in the guinea pig auditory epithelium. Sci. Rep. 7:46058. 10.1038/srep46058 28387239 PMC5384248

[B44] Li X. Bi Z. Sun Y. Li C. Li Y. Liu Z. (2020). *In vivo* ectopic Ngn1 and Neurod1 convert neonatal cochlear glial cells into spiral ganglion neurons. FASEB J. 34, 4764–4782. 10.1096/fj.201902118R 32027432

[B43] Li C. Li X. Bi Z. Sugino K. Wang G. Zhu T.. (2020). Comprehensive transcriptome analysis of cochlear spiral ganglion neurons at multiple ages. eLife 9:e50491. 10.7554/eLife.50491 31913118 PMC7299348

[B45] Li X. Shi X. Wang C. Niu H. Zeng L. Qiao Y. (2016). Cochlear spiral ganglion neuron apoptosis in neonatal mice with murine cytomegalovirus-induced sensorineural hearing loss. J. Am. Acad. Audiol. 27, 345–353. 10.3766/jaaa.15061 27115244

[B46] Liberman M. C. (2017). Noise-induced and age-related hearing loss: new perspectives and potential therapies. F1000Res. 6:927. 10.12688/f1000research.11310.1 28690836 PMC5482333

[B47] Liu W. Glueckert R. Linthicum F. H. Rieger G. Blumer M. Bitsche M.. (2014). Possible role of gap junction intercellular channels and connexin 43 in satellite glial cells (SGCs) for preservation of human spiral ganglion neurons : a comparative study with clinical implications. Cell Tissue Res. 355, 267–278. 10.1007/s00441-013-1735-2 24241398 PMC3921454

[B50] Liu Y. Qi J. Chen X. Tang M. Chu C. Zhu W.. (2019). Critical role of spectrin in hearing development and deafness. Sci. Adv. 5:eaav7803. 10.1126/sciadv.aav7803 31001589 PMC6469942

[B51] Liu Z. Tang M. Zhao J. Chai R. Kang J. (2018). Looking into the future: toward advanced 3D biomaterials for stem-cell-based regenerative medicine. Adv. Mater. 30:e1705388. 10.1002/adma.201705388 29450919

[B48] Liu W. Xu L. Wang X. Zhang D. Sun G. Wang M.. (2021). PRDX1 activates autophagy *via* the PTEN-AKT signaling pathway to protect against cisplatin-induced spiral ganglion neuron damage. Autophagy 17, 4159–4181. 10.1080/15548627.2021.1905466 33749526 PMC8726717

[B49] Liu W. Xu X. Fan Z. Sun G. Han Y. Zhang D.. (2019). Wnt signaling activates TP53-induced glycolysis and apoptosis regulator and protects against cisplatin-induced spiral ganglion neuron damage in the mouse cochlea. Antioxid. Redox Signal. 30, 1389–1410. 10.1089/ars.2017.7288 29587485

[B52] Lv J. Fu X. Li Y. Hong G. Li P. Lin J.. (2021). Deletion of Kcnj16 in mice does not alter auditory function. Front. Cell Dev. Biol. 9:630361. 10.3389/fcell.2021.630361 33693002 PMC7937937

[B53] McLean W. J. McLean D. T. Eatock R. A. Edge A. S. (2016). Distinct capacity for differentiation to inner ear cell types by progenitor cells of the cochlea and vestibular organs. Development 143, 4381–4393. 10.1242/dev.139840 27789624 PMC5201044

[B54] Nishimura K. Nakagawa T. Sakamoto T. Ito J. (2012). Fates of murine pluripotent stem cell-derived neural progenitors following transplantation into mouse cochleae. Cell Transplant. 21, 763–771. 10.3727/096368911X623907 22305181

[B55] Nishimura K. Weichert R. M. Liu W. Davis R. L. Dabdoub A. (2014). Generation of induced neurons by direct reprogramming in the mammalian cochlea. Neuroscience 275, 125–135. 10.1016/j.neuroscience.2014.05.067 24928351

[B56] Noda T. Meas S. J. Nogami J. Amemiya Y. Uchi R. Ohkawa Y.. (2018). Direct reprogramming of spiral ganglion non-neuronal cells into neurons: toward ameliorating sensorineural hearing loss by gene therapy. Front. Cell Dev. Biol. 6:16. 10.3389/fcell.2018.00016 29492404 PMC5817057

[B57] Nyberg S. Abbott N. J. Shi X. Steyger P. S. Dabdoub A. (2019). Delivery of therapeutics to the inner ear: the challenge of the blood-labyrinth barrier. Sci. Transl. Med. 11:eaao0935. 10.1126/scitranslmed.aao0935 30842313 PMC6488020

[B58] Pekny M. Wilhelmsson U. Pekna M. (2014). The dual role of astrocyte activation and reactive gliosis. Neurosci. Lett. 565, 30–38. 10.1016/j.neulet.2013.12.071 24406153

[B59] Perny M. Ting C. C. Kleinlogel S. Senn P. Roccio M. (2017). Generation of otic sensory neurons from mouse embryonic stem cells in 3D culture. Front. Cell. Neurosci. 11:409. 10.3389/fncel.2017.00409 29311837 PMC5742223

[B60] Pinyon J. L. Tadros S. F. Froud K. E. AC Y. W. Tompson I. T. Crawford E. N.. (2014). Close-field electroporation gene delivery using the cochlear implant electrode array enhances the bionic ear. Sci. Transl. Med. 6:233ra254. 10.1126/scitranslmed.3008177 24760189

[B61] Rask-Andersen H. Bostrom M. Gerdin B. Kinnefors A. Nyberg G. Engstrand T.. (2005). Regeneration of human auditory nerve. *in vitro*/in video demonstration of neural progenitor cells in adult human and guinea pig spiral ganglion. Hear. Res. 203, 180–191. 10.1016/j.heares.2004.12.005 15855043

[B62] Reyes J. H. O’Shea K. S. Wys N. L. Velkey J. M. Prieskorn D. M. Wesolowski K.. (2008). Glutamatergic neuronal differentiation of mouse embryonic stem cells after transient expression of neurogenin 1 and treatment with BDNF and GDNF: *in vitro* and *in vivo* studies. J. Neurosci. 28, 12622–12631. 10.1523/JNEUROSCI.0563-08.2008 19036956 PMC2729437

[B63] Rousset F. V B. C. K. Sipione R. Schmidbauer D. Nacher-Soler G. Ilmjarv S.. (2020). Intrinsically self-renewing neuroprogenitors from the A/J mouse spiral ganglion as virtually unlimited source of mature auditory neurons. Front. Cell. Neurosci. 14:395. 10.3389/fncel.2020.599152 33362466 PMC7761749

[B64] Scheper V. Hoffmann A. Gepp M. M. Schulz A. Hamm A. Pannier C.. (2019). Stem cell based drug delivery for protection of auditory neurons in a guinea pig model of cochlear implantation. Front. Cell. Neurosci. 13:177. 10.3389/fncel.2019.00177 31139049 PMC6527816

[B65] Schulze J. Staecker H. Wedekind D. Lenarz T. Warnecke A. (2020). Expression pattern of brain-derived neurotrophic factor and its associated receptors: implications for exogenous neurotrophin application. Hear. Res. 10.1016/j.heares.2020.108098. [Online ahead of print]. 33143996

[B66] Shrestha B. R. Chia C. Wu L. Kujawa S. G. Liberman M. C. Goodrich L. V. (2018). Sensory neuron diversity in the inner ear is shaped by activity. Cell 174, 1229–1246.e17. 10.1016/j.cell.2018.07.007 30078709 PMC6150604

[B67] Sun S. Babola T. Pregernig G. So K. S. Nguyen M. Su S.-S. M.. (2018). Hair cell mechanotransduction regulates spontaneous activity and spiral ganglion subtype specification in the auditory system. Cell 174, 1247–1263.e15. 10.1016/j.cell.2018.07.008 30078710 PMC6429032

[B68] Tasdemir-Yilmaz O. E. Druckenbrod N. R. Olukoya O. O. Dong W. Yung A. R. Bastille I.. (2021). Diversity of developing peripheral glia revealed by single-cell RNA sequencing. Dev. Cell 56, 2516–2535.e8. 10.1016/j.devcel.2021.08.005 34469751 PMC8442251

[B70] Wang Q. Green S. H. (2011). Functional role of neurotrophin-3 in synapse regeneration by spiral ganglion neurons on inner hair cells after excitotoxic trauma *in vitro*. J. Neurosci. 31, 7938–7949. 10.1523/JNEUROSCI.1434-10.2011 21613508 PMC3132175

[B69] Wang M. Han Y. Wang X. Liang S. Bo C. Zhang Z.. (2021). Characterization of EGR-1 expression in the auditory cortex following kanamycin-induced hearing loss in mice. J. Mol. Neurosci. 71, 2260–2274. 10.1007/s12031-021-01791-0 33423191

[B72] Wei H. Chen Z. Hu Y. Cao W. Ma X. Zhang C.. (2021). Topographically conductive butterfly wing substrates for directed spiral ganglion neuron growth. Small 17:e2102062. 10.1002/smll.202102062 34411420

[B71] Wei D. Jin Z. Järlebark L. Scarfone E. Ulfendahl M. (2007). Survival, synaptogenesis and regeneration of adult mouse spiral ganglion neurons*in vitro*. J. Neurobiol. 67, 108–122. 10.1002/dneu.20336 17443776

[B73] Wise A. K. Pujol R. Landry T. G. Fallon J. B. Shepherd R. K. (2017). Structural and ultrastructural changes to type I spiral ganglion neurons and schwann cells in the deafened guinea pig cochlea. J. Assoc. Res. Otolaryngol. 18, 751–769. 10.1007/s10162-017-0631-y 28717876 PMC5688041

[B74] Xia L. Shang Y. Chen X. Li H. Xu X. Liu W.. (2020). Oriented neural spheroid formation and differentiation of neural stem cells guided by anisotropic inverse opals. Front. Bioeng. Biotechnol. 8:848. 10.3389/fbioe.2020.00848 32850719 PMC7411081

[B75] Yan W. Liu W. Qi J. Fang Q. Fan Z. Sun G.. (2018). A three-dimensional culture system with matrigel promotes purified spiral ganglion neuron survival and function *in vitro*. Mol. Neurobiol. 55, 2070–2084. 10.1007/s12035-017-0471-0 28283883

[B76] Yuan Y. Shi F. Yin Y. Tong M. Lang H. Polley D. B.. (2013). Ouabain-induced cochlear nerve degeneration: synaptic loss and plasticity in a mouse model of auditory neuropathy. J. Assoc. Res. Otolaryngol. 15, 31–43. 10.1007/s10162-013-0419-7 24113829 PMC3901858

[B80] Zhang Z. J. Guan H. X. Yang K. Xiao B. K. Liao H. Jiang Y.. (2018). Estimation of the status of spiral ganglion neurons and Schwann cells in the auditory neural degeneration mouse using the auditory brainstem response. Acta Otolaryngol. 138, 603–609. 10.1080/00016489.2018.1436766 29553844

[B78] Zhang Y. He Q. Dong J. Jia Z. Hao F. Shan C. (2016). Effects of epigallocatechin-3-gallate on proliferation and differentiation of mouse cochlear neural stem cells: involvement of PI3K/Akt signaling pathway. Eur. J. Pharm. Sci. 88, 267–273. 10.1016/j.ejps.2016.03.017 27012759

[B77] Zhang P. Z. He Y. Jiang X. W. Chen F. Q. Chen Y. Shi L.. (2013). Stem cell transplantation *via* the cochlear lateral wall for replacement of degenerated spiral ganglion neurons. Hear. Res. 298, 1–9. 10.1016/j.heares.2013.01.022 23403006

[B79] Zhang Y. Li Y. Fu X. Wang P. Wang Q. Meng W.. (2021). The detrimental and beneficial functions of macrophages after cochlear injury. Front. Cell Dev. Biol. 9:631904. 10.3389/fcell.2021.631904 34458249 PMC8385413

[B81] Zhou H. Qian X. Xu N. Zhang S. Zhu G. Zhang Y.. (2020). Disruption of Atg7-dependent autophagy causes electromotility disturbances, outer hair cell loss and deafness in mice. Cell Death Dis. 11:913. 10.1038/s41419-020-03110-8 33099575 PMC7585579

[B82] Zong L. Chen K. Zhou W. Jiang D. Sun L. Zhang X.. (2014). Inner ear stem cells derived feeder layer promote directional differentiation of amniotic fluid stem cells into functional neurons. Hear. Res. 316, 57–64. 10.1016/j.heares.2014.07.012 25124154

